# Supplementary material for: DMRforPairs: identifying Differentially Methylated Regions between unique samples using array based methylation profiles
Source: BMC Bioinformatics. 2014 May 15;15:141. doi: 10.1186/1471-2105-15-141 (PMC4046028; doi:10.1186/1471-2105-15-141)
Supplement: Additional file 2 — DMRforPairs output for the comparison of A431-MCF7 and NA17018-NA17105. Please start from the HTML files in each folder. Available via the BMC Bioinformatics website. [file 1471-2105-15-141-S2.zip › 1394847754114233_MOESM2_ESM/A431_MCF7/figures/7402.pdf]

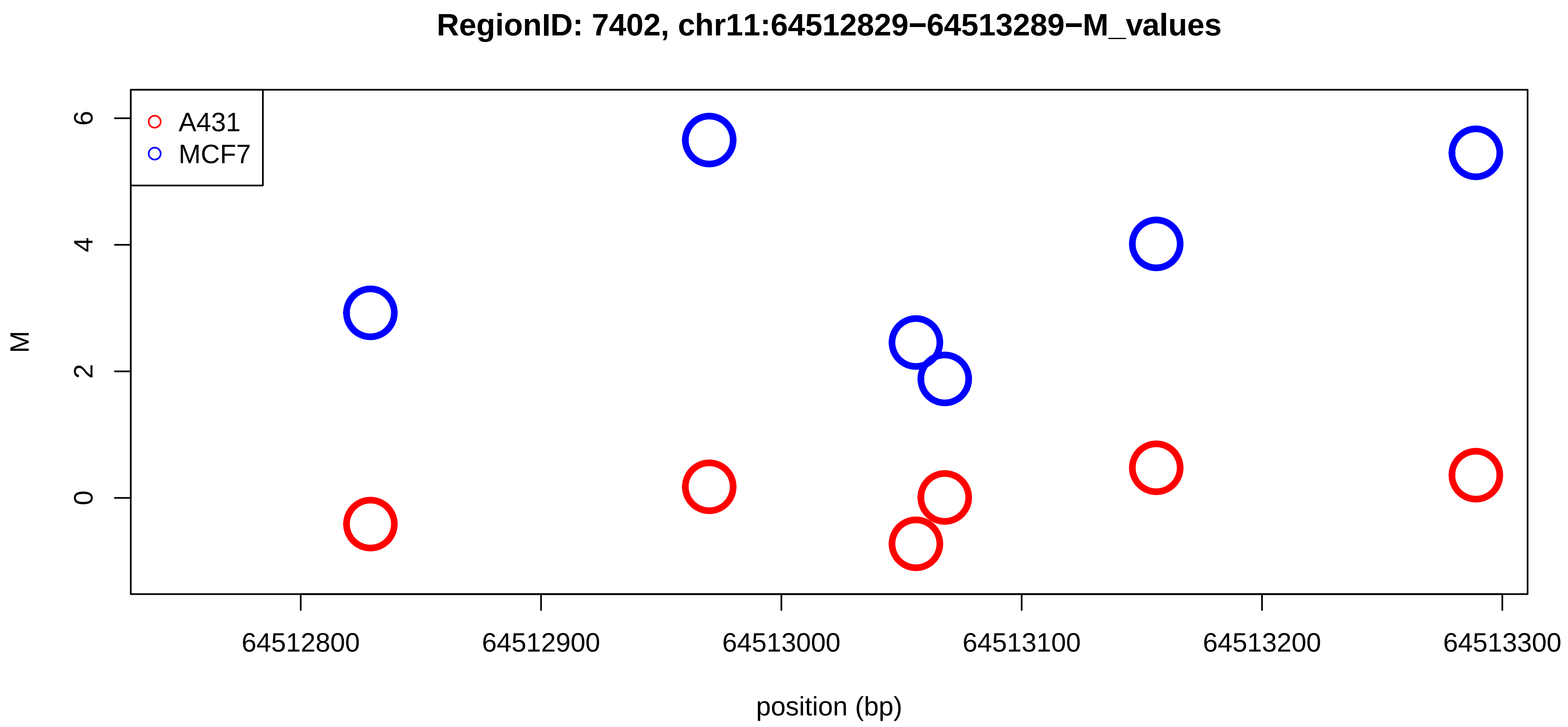

RegionID: 7402, chr11:64512829−64513289−M_values
A431
MCF7
M
0
2
4
6
64512800
64512900
64513000
64513100
64513200
64513300
position (bp)

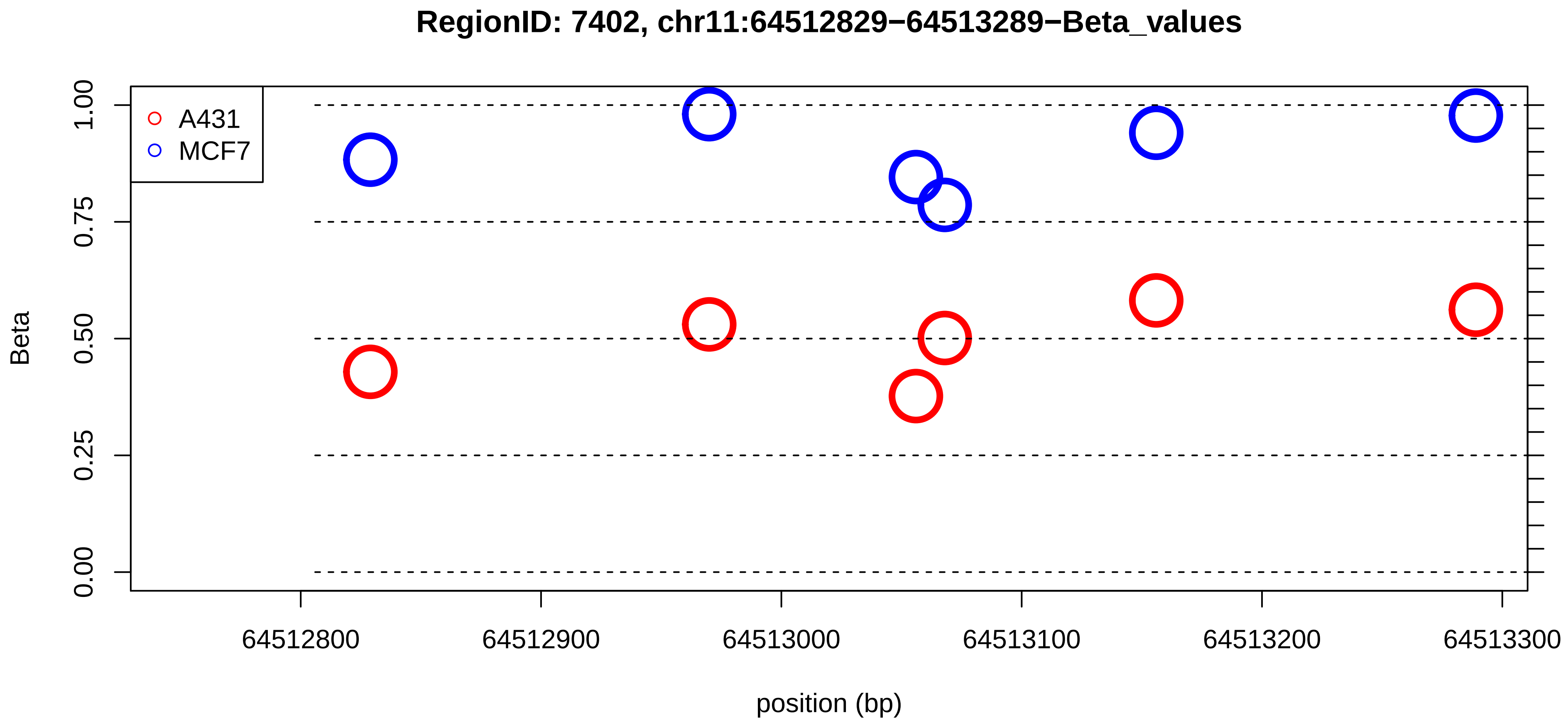

RegionID: 7402, chr11:64512829−64513289−Beta_values
A431
MCF7
Beta
0.00
0.25
0.50
0.75
1.00
64512800
64512900
64513000
64513100
64513200
64513300
position (bp)

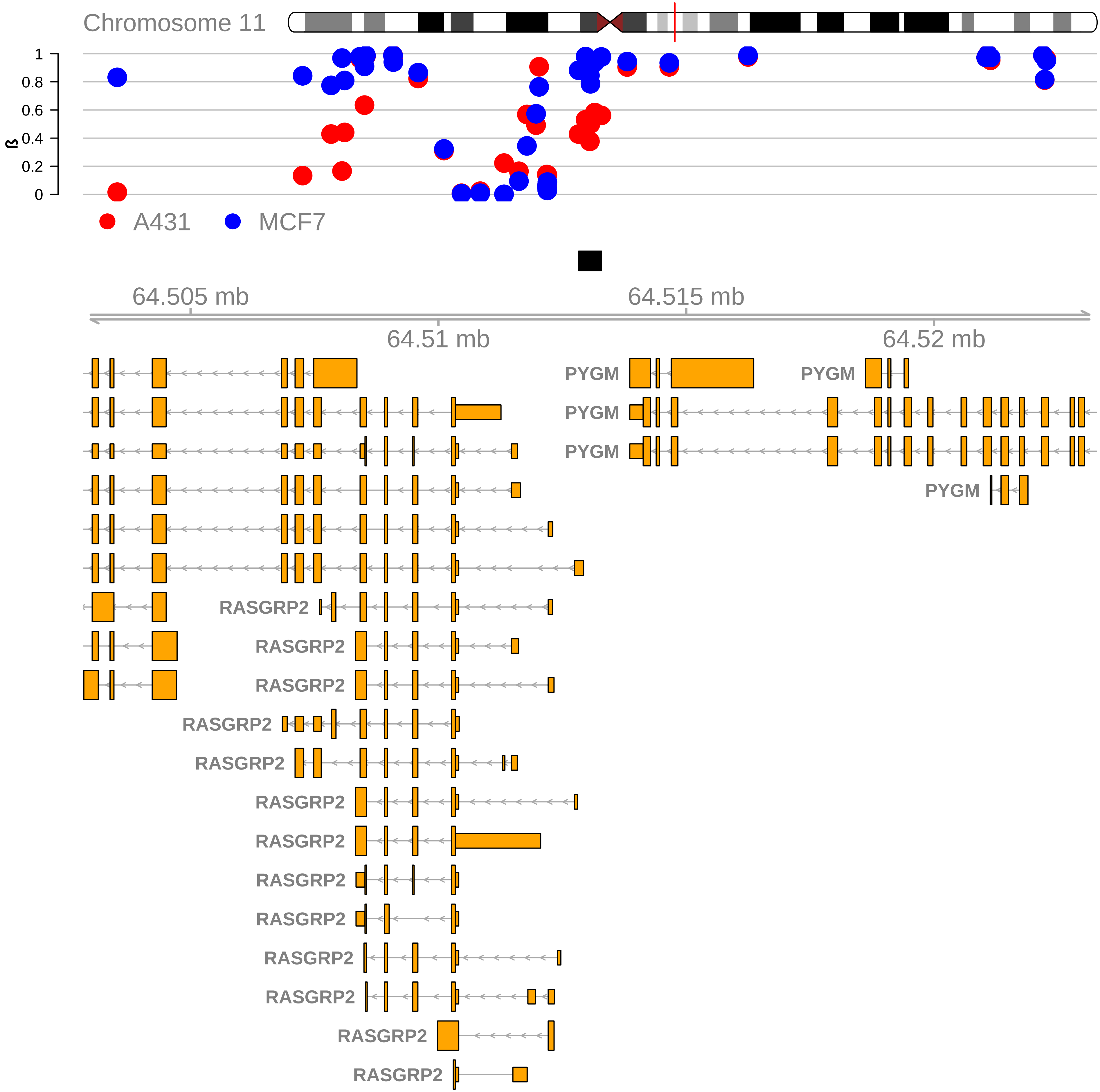

Chromosome 11
ß
1
0.8
0.6
0.4
0.2
0
A431
MCF7
64.505 mb
64.515 mb
64.51 mb
64.52 mb
PYGM
PYGM
PYGM
PYGM
PYGM
RASGRP2
RASGRP2
RASGRP2
RASGRP2
RASGRP2
RASGRP2
RASGRP2
RASGRP2
RASGRP2
RASGRP2
RASGRP2
RASGRP2
RASGRP2
